# Association Between Angina Symptom Characteristics and Obstructive Coronary Artery Disease: A Comparative Cross-Sectional Study

**DOI:** 10.1097/jnr.0000000000000693

**Published:** 2025-08-04

**Authors:** Ching-Ching TSAI, I-Chang HSIEH, Pao-Hsien CHU, Ming-Jer HSIEH, Hsin-Fu LEE, Lun-Hui HO

**Affiliations:** 1 Department of Nursing, College of Nursing, Chang Gung University of Science and Technology, Taoyuan, Taiwan, ROC; 2 Department of Cardiology, Chang Gung Memorial Hospital, Linkou, Taiwan, ROC; 3 Department of Cardiology, Heart Failure Center, Chang Gung Memorial Hospital, Linkou, Taiwan, ROC; 4 College of Medicine, Chang Gung University, Taoyuan, Taiwan, ROC; 5 Graduate Institute of Clinical Medical Sciences, College of Medicine, Chang Gung University, Taoyuan, Taiwan, ROC; 6 Department of Cardiology, New Taipei City Municipal Tucheng Hospital (Chang Gung Memorial Hospital, Tucheng), Tucheng, Taiwan, ROC; 7 Department of Nursing Management of the Administration Center, Chang Gung Medical Foundation, Taiwan, ROC; † Contributed equally

**Keywords:** angina symptoms, coronary artery disease, risk assessment, predictive models

## Abstract

**Background:**

Angina, the primary symptom reported by patients with coronary artery disease (CAD), is an essential consideration in CAD treatment strategies and evaluations of treatment efficacy. However, research exploring the relationship between angina symptom characteristics (severity, type, and frequency) and obstructive CAD is scarce.

**Purpose:**

This study was designed to investigate the association between obstructive CAD and angina symptom severity, type, and frequency.

**Methods:**

A cross-sectional approach was used to analyze baseline data from two studies conducted from March 2018 to September 2021 at a medical center in northern Taiwan. The sample comprised 723 patients undergoing invasive coronary angiography for suspected or known CAD. The participants were categorized into obstructive (≥50% stenosis in at least one epicardial artery) and control groups. Angina symptom severity, type, and frequency were assessed using the Angina Evaluation Form.

**Results:**

The average age of the participants was 60.1 years, and most (83.7%) were male. Nearly three-quarters (73.1%) had Class 1 or 2 angina, 60.3% reported experiencing typical angina, and 46% had angina episodes at least once per week. The results of multiple logistic regression analysis found older age, male sex, history of diabetes, currently smoking, higher low-density lipoprotein levels, lower estimated glomerular filtration rate, and typical angina to be associated with a higher risk of obstructive CAD. Risk of obstructive CAD was twice as high in participants experiencing typical angina symptoms than those experiencing atypical angina symptoms (*OR*=1.97, 95% CI=[1.09, 3.54], *p*=.025). Subgroup analysis findings identified associations to be higher in younger (*OR*=2.22, 95% CI=[1.23, 3.99], *p*=.008) and male (*OR*=1.83, 95% CI=[1.08, 3.09], *p*=.024) participants. Also, the risk of obstructive CAD was higher for those experiencing angina episodes less than twice per week, only in the subgroup of older individuals. No significant relationship between angina symptom severity and risk of obstructive CAD was found.

**Conclusions/Implications for Practice:**

Angina type, rather than severity or frequency, is significantly associated with obstructive CAD. The findings of this study emphasize the importance of conducting comprehensive angina symptom evaluation in risk assessments on patients with suspected or known CAD. Furthermore, large-scale prospective studies should be conducted in different population groups and settings to validate angina symptoms as a predictor of obstructive CAD.

## Introduction

Coronary artery disease (CAD), a leading cause of death worldwide as well as in Taiwan (Ueng et al., 2023), affects the epicardial coronary arteries and may lead to myocardial ischemia due to risk factors such as family history, hypertension, diabetes, dyslipidemia, obesity, personal attributes (e.g., smoking, lack of exercise, psychological stress), and atherosclerosis (Ueng et al., 2023). The International Consortium for Health Outcomes Measurement recommends monitoring patient-reported outcomes, specifically angina, in patients with CAD for up to 5 years after the initial event (McNamara et al., 2015). Angina is a common symptom observed in both obstructive and nonobstructive CAD. However, perceptions of angina are subjective and may be influenced by biological, cognitive, behavioral, environmental, and other factors (Zimmerman et al., 2016). Therefore, medical staff must be able to differentiate angina-like symptoms caused by non-CAD conditions such as heart disease, gastrointestinal disease, and psychological disorders (Knuuti et al., 2020; C. C. Tsai, Chuang, et al., 2019) from those caused by CAD. Despite the considerable attention given to the relationship between angina symptoms and CAD (Nakas et al., 2019), many uncertainties and gaps in knowledge remain.

First, the Diamond and Forrester model, which considers age, sex, and angina symptoms, tends to overestimate the prevalence of obstructive CAD (Reeh et al., 2019). Although subsequent attempts have been made to improve the accuracy of this model by adding traditional risk factors like diabetes, hypertension, dyslipidemia, obesity, family history, and smoking (Baskaran et al., 2020; Nakas et al., 2019; Reeh et al., 2019), the influence on CAD of personal attributes such as diet, exercise, and psychosocial factors has yet to be clarified in the literature. Second, the preponderance of prior research has focused on correlations between different types of angina (i.e., typical angina, atypical angina, and nonanginal chest pain) and CAD (Madsen et al., 2017; Nakas et al., 2019; Peng et al., 2021), with few studies designed to explore the relationship between angina symptom severity/frequency and CAD (Baskaran et al., 2020; Guimaraes et al., 2021). Notably, comprehensive investigations into the concurrent impact of various angina characteristics, for example, severity, type, and frequency, on CAD are lacking. Third, several studies have established a link between typical angina symptoms and an elevated risk of obstructive CAD (Madsen et al., 2017; Nakas et al., 2019; Peng et al., 2021), with asymptomatic or atypical angina identified as predictors of nonobstructive CAD (stenosis <50%; Peerwani et al., 2023). The Canadian Cardiovascular Society Classification (CCSC) employs a grading system based on physical activity thresholds to define angina severity (Ueng et al., 2023). Patients classified as CCSC class III/IV (higher symptom severity) were found to have higher SYNTAX, Gensini, and Friesinger atherosclerosis burden scores than asymptomatic patients (Guimaraes et al., 2021). In addition, having a high baseline average peak velocity has been shown to suggest lower coronary flow reserve and potentially worse angina frequency (Suppogu et al., 2021). However, other studies have found no correlation between angina symptoms and obstructive CAD, reporting angina symptoms to have limited value in predicting CAD (Gur, 2019; Mehta et al., 2019; Rovai et al., 2015). Fourth and finally, angina is a subjective symptom influenced by culture and ethnic background (Zimmerman et al., 2016). Significant differences in chest pain presentation have been reported between different populations, for example, Europeans and East Asians (Greenslade et al., 2012; King-Shier et al., 2019). For example, typical angina symptoms have been identified as a predictor of acute coronary syndrome in Indian patients but not in European, Chinese, or Korean patients (Greenslade et al., 2012). However, few studies examining the relationship between angina symptoms and obstructive CAD specifically in the Taiwanese population have been conducted.

Clinical guidelines generally direct medical staff to conduct a comprehensive assessment of angina symptoms as the first step in dealing with patients who may have CAD (Knuuti et al., 2020; Gulati et al., 2021). Assessment tools for angina symptoms should address factors that provoke and relieve the pain, identify the location and radiation of the pain, determine pain severity, determine the timing and cause of onset, and determine the duration of the pain (Kwon & Lee, 2023). The Rose Angina Questionnaire, CCSC, and Seattle Angina Questionnaire (SAQ) are commonly used to measure the specific aspects of angina symptoms (Haider et al., 2020). In Taiwan, the CCSC has been used to grade the severity of angina symptoms in patients with CAD (Huang et al., 2017), while the Rose Angina Questionnaire has been used to investigate the prevalence of angina symptoms both in specific settings (Chen et al., 1996; Lin et al., 2004) and nationwide (C. C. Tsai, Hsieh, et al., 2019). However, the lack of an assessment instrument empirically validated for the Taiwanese population indicates the lack of appropriate tools to effectively assess angina symptoms in Taiwan.

Given that existing angina symptom assessment tools cannot evaluate severity, type, and frequency simultaneously, in this study, a reliable and validated tool was designed to assess angina symptoms in patients with CAD. The main objective of this study was to investigate the relationship between various angina symptom characteristics and obstructive CAD identified by invasive coronary angiography (ICA) in Taiwan. Severity, type, and frequency of angina symptoms were each hypothesized to be significantly associated with obstructive CAD.

## Methods

### Study Design and Participant Selection

A cross-sectional design combining baseline data from 2 previous studies was used in this study. The first study spanned March 2018 to March 2019 and aimed to develop and validate an assessment form for multiple symptoms of CAD. The second study spanned August 2019 to September 2021 and aimed to compare differences in essential characteristics among symptom clusters while exploring the impact of symptom clusters on quality of life, disease severity, and prognosis.

Both of the studies were approved by the Chang Gung Medical Foundation institutional review board (No. 201601502B0 and 201801810B0). All of the participants provided written informed consent before participation. This study included inpatients who had undergone ICA at a medical center in northern Taiwan, were at least 21 years old, and could communicate effectively. Otherwise, eligible patients affected by psychiatric disorders, end-stage renal disease, or other systemic disorders (e.g., severe infection, trauma, surgery, cancer, other major diseases) within the preceding 3 months were excluded. Of the 1,477 patients initially screened, 222 were excluded due to communication difficulties, and another 249 met at least one of the exclusion criteria. Of the 1,006 eligible participants, 283 refused to participate, resulting in a study sample of 723 (participation rate: 71.9%). No significant differences in either age or sex were found between participants and nonparticipants (*p*>.05).

The results of a study conducted in China by Peng et al. (2021) indicate that patients with typical angina symptoms face a 1.65 times higher risk of obstructive CAD (stenosis ≥50% by ICA) than their peers with atypical angina symptoms. To ensure adequate statistical power for this study, the G Power 3.1.9.4 software estimated a sample size of 514 people for a two-tailed logistic regression analysis, with a power of 0.8 and an α of .05.

### Angina Assessment

The Angina Evaluation Form (AEF) was developed for this study to evaluate angina symptoms in patients with CAD by referring to clinical experience and commonly used tools. The AEF consists of seven questions and assesses three characteristics of angina: severity, type, and frequency (Appendix, Supplemental Digital Content 1, http://links.lww.com/JNR/A6).

The first question (Q1) is as follows: “Do you ever experience chest pain/discomfort?” If the answer is “no,” the respondent is considered asymptomatic. If the answer is “yes,” the respondent answers the remaining six questions, as follows: Q2 (“In which of the following situations do you usually feel chest pain/discomfort?”) uses the CCSC as a reference to determine the severity of angina based on classes 0–4, with class 0 indicating no symptoms and subsequent classes indicating increasing severity (Kemp et al., 2019; Knuuti et al., 2020; Ueng et al., 2023). Q3–Q6 assess alleviating factors, duration, and chest pain/discomfort site. The respondent is asked, “What do you usually do when you feel chest pain/discomfort while walking?” (Q3); “When you feel chest pain/discomfort while walking: if you stop moving can you usually make this pain/discomfort disappear?” (Q4); “When you feel chest pain/discomfort, how long does it usually take to disappear?” (Q5); and “Where does your chest pain/discomfort usually occur?” (i.e., left upper chest, left lower chest, middle-upper chest, middle-lower chest, right upper chest, and/or right lower chest; Q6). To demonstrate “typical” angina, respondents were required to (a) demonstrate symptoms provoked by exertion (Q2, CCSC Class 1–3); (b) slow or stop moving when pain/discomfort occurs (Q3); (c) experience cessation of pain/discomfort after stopping (Q4); (d) have symptoms that lasted <10 minutes (Q5); and (e) report pain/discomfort at least one specific site in the left, center, upper, and lower chest (Q6; Rahman et al., 2013). Q7 was “How often did you feel chest pain/discomfort in the past month?”, with a 5-point Likert scale used to score frequency, with higher scores indicating more frequent angina.

Initially, we invited 10 cardiology experts (three attending physicians, two head nurses, two nurse practitioners, and three nursing teachers) to score the universality, clarity, and applicability of each question in the original AEF using a 4-point Likert scale. The content validity index (CVI) of all items was between 0.8 and 1.0, while the overall CVI was .91, indicating the AEF had good content validity (Almanasreh et al., 2019). To test the feasibility of the AEF, a pilot study was conducted with 10 patients with CAD selected via convenience sampling. After revision, the AEF was used for testing.

The validity and reliability of the AEF were previously examined on 346 patients. The test–retest correlation coefficient over 1–2 days was .86–1.0, with an overall mean of .93, indicating excellent reproducibility (Chan et al., 2014; Li et al., 2014). When compared with the Chinese version of the Seattle Angina Questionnaire (SAQ-C) as a criterion (M. W. Tsai & Chie, 2002), the AEF showed a slightly negative correlation with the physical limitation subscale in the SAQ-C (*r*=−.22, *p* < .001) for severity, while frequency assessed by the AEF demonstrated a high negative correlation with angina stability and frequency subscale scores in the SAQ-C (*r*=−.69, angina stability; −.9, angina frequency; *p*<.001; Huber et al., 2020).

### Additional Variables Considered

A basic information form was used to collect the demographic, medical history, and personal attribute data from participants, used to evaluate risk factors for CAD. The participants were asked if they had undergone cardiac catheterization and/or revascularization procedures such as coronary angioplasty, stenting, and coronary artery bypass grafting (CABG). Disease history information, including myocardial infarction, diabetes, and hypertension, was based on self-reported doctor-diagnosed conditions. Those participants with a family history of CAD, i.e., having grandparents, parents, or siblings who had developed CAD before age 60, were considered to have a positive family history of premature CAD. Current smokers were defined as individuals who had smoked over 100 cigarettes in their lifetime and either quit smoking for less than one year or were currently smoking. Exposure to secondhand smoke was determined by whether participants had encountered someone smoking at home or in the workplace during the past week. Physical activity was defined as regular exercise outside of work, daily life, and housework during the preceding two weeks. The participants were also evaluated on their alcohol consumption during the past 2 weeks and regular use of dietary supplements over the past 12 months (C. C. Tsai, Hsieh, et al., 2019).

Also, the dietary habits of the participants over the past two weeks were assessed. The frequency of following a heart-healthy diet (low fat/cholesterol, low calorie, low sodium, low sugar) was scored on a scale of 0–4, where 0 means “*none*” and 4 means “*always.*” The total score ranged from 0 to 16, with higher scores indicating healthier dietary habits. The internal consistency of the scoring system was high, with a Cronbach’s α of .97. Psychological distress was measured using the Brief Symptom Rating Scale (BSRS-5), which uses a Likert-like scale ranging from 0-20 points, with scores of 6 or higher indicating psychological distress. The BSRS-5 is considered valid and reliable in Taiwan (Lee et al., 2003), with a Cronbach’s α of .89. Economic status was determined using a visual analog scale (0-10). Three items were used to evaluate social support level, with two assessing perceived support from family members and relatives, while the third evaluating overall satisfaction with support. Each item was scored from 0 to 10, with the total possible score ranging from 0 to 30 and higher scores indicating higher levels of social support. The internal consistency of the scoring system for social support was high, with a Cronbach’s α of .93.

The medical history data extracted and used in this study included the results of cardiac-related examinations, laboratory tests, the diagnosis at admission, and medications used for CAD at admission. Blood pressure, height, and weight were recorded at admission, and the body mass index was calculated as weight (kg) divided by height (m^2^; C. C. Tsai, Hsieh et al., 2019). A positive stress test result on the exercise electrocardiography and/or myocardial perfusion imaging was indicative of ischemia or infarction. Left ventricular ejection fraction (LVEF) and either wall motion hypokinesia or dyskinesis were considered abnormal findings. Examinations or measurements conducted at outpatient or emergency departments before hospitalization were not repeated unless necessary. Therefore, all of the examinations were performed within 3 months before and during hospitalization. The test result closest to the ICA date was used.

### Invasive Coronary Angiography

If multiple angiograms were conducted during the current hospitalization, the most severe outcome was considered. Obstructive CAD was defined as the narrowing of ≥50% in any of the epicardial coronary arteries, including the left main trunk, left anterior descending, left circumflex, and right coronary artery. Significant obstruction (stenosis of ≥50%) in the left main trunk was considered indicative of two-vessel stenosis (McNamara et al., 2015); otherwise, one-vessel disease was suggested. Percutaneous coronary intervention or CABG was regarded as equivalent to having undergone a revascularization procedure (Baskaran et al., 2020).

### Data Collection Process

Data were collected in the same manner for both of the included studies. The participants completed the essential information and the AEF by themselves. Those who were illiterate or unable to complete the forms were asked the questions orally by the research assistant, who recorded their responses. All questions took a total of around 15–20 minutes to answer. After patient discharge, the investigator extracted data from their medical records. The funder of this research did not participate in the study or manuscript preparation.

### Data Analysis Approach

The data were analyzed using SPSS version 26.0 (IBM Corp., Armonk, NY, USA). Categorical data were presented as numbers and percentages. Normally distributed continuous data, with absolute skewness and kurtosis values of <2 (Siu et al., 2024), were reported as mean and standard deviation (*SD)*, whereas non-normally distributed data were reported as medians and interquartile ranges. Asymptomatic participants were classified as having infrequent, atypical symptoms. Simple logistic regression was used to examine the association between candidate factors and significant coronary artery obstruction. Variables with a significance level of *p*<.05 were entered into a stepwise regression analysis to identify factors associated with obstructive CAD (Peng et al., 2021), with results expressed as odds ratios (*OR*) with 95% confidence intervals (CI). Subgroup analyses based on age and sex were also performed, considering the small number of female participants and the variability of angina symptom presentation and predictive value for obstructive CAD based on age and sex (Knuuti et al., 2020). All of the tests were two-tailed, with *p*<.05 considered statistically significant.

## Results

### Basic Characteristics of Participants

The 723 participants enrolled in the study had an average age of 60.1 years. Most (83.7%) were male, 73.1% reported class 1 or 2 angina severity, 60.3% had typical angina, and 46% experienced angina episodes at least once a week (Table 1). The obstructive group (*n*=545; 75.4%) showed significantly worse LVEF than the control group (*n*=178; 24.6%; Table 2).

**Table 1:** Risk Factors of Coronary Artery Disease: Simple Logistic Regression Analysis

Variable	All (*n*=723)	Control (*n*=178)	Obstructive (*n*=545)	*p*
	*n* (%)/*M*±*SD*	*n* (%)/*M*±*SD*	*n* (%)/*M*±*SD*	
**Demographic data**				
Age (year)	60.1±10.3	58.1±11.0	60.7±10.0	.003**
Sex				
Female	118 (16.3)	42 (23.6)	76 (13.9)	—
Male	605 (83.7)	136 (76.4)	469 (86.1)	.003**
Married with a living spouse	594 (82.5)	145 (81.5)	449 (82.4)	.780
Have children	635 (87.8)	152 (85.4)	483 (88.6)	.254
Living with family	660 (91.3)	164 (92.1)	496 (91.0)	.644
Educational level								
None or elementary school	148 (20.5)	34 (19.1)	114 (20.9)	—
Middle or high school	362 (50.1)	84 (47.2)	278 (51.0)	.955
College or higher	213 (29.5)	60 (33.7)	153 (28.1)	.269
Currently working	388 (53.7)	102 (57.3)	286 (52.5)	.263
**Past medical history (yes)**				
Cardiac catheterization	275 (38.0)	58 (32.6)	217 (39.8)	.085
Revascularization procedures	242 (33.5)	47 (26.4)	195 (35.8)	.022*
Myocardial infarction	127 (17.6)	26 (14.6)	101 (18.5)	.233
Diabetes	230 (31.8)	34 (19.1)	196 (36.0)	<.001**
Hypertension	399 (55.2)	96 (53.9)	303 (55.6)	.698
Dyslipidemia	239 (33.1)	50 (28.1)	189 (34.7)	.105
Stroke	42 (5.8)	6 (3.4)	36 (6.6)	.116
Renal disease	27 (3.7)	6 (3.4)	21 (3.9)	.768
Respiratory disease	18 (2.5)	7 (3.9)	11 (2.0)	.162
Digestive disease	105 (14.5)	32 (18.0)	73 (13.4)	.133
Thyroid disease	10 (1.4)	3 (1.7)	7 (1.3)	.692
Peripheral arterial occlusion disease	7 (1.0)	0	7 (1.3)	.999
Gout	51 (7.1)	9 (5.1)	42 (7.7)	.234
Anemia	2 (0.3)	1 (0.6)	1 (0.2)	.428
Psychological disease	11 (1.5)	7 (3.9)	4 (0.7)	.007**
**Personal attributes**				
Family history of premature coronary artery disease (yes)	234 (32.4)	47 (26.4)	187 (34.3)	.051
Current smoker (yes)	222 (30.7)	42 (23.6)	180 (33.0)	.018*
Secondhand smoke exposure (yes)	156 (21.6)	32 (18.0)	124 (22.8)	.180
Physical activity (yes)	207 (28.6)	50 (28.1)	157 (28.2)	.854
Alcohol consumption (yes)	76 (10.5)	17 (9.6)	59 (10.8)	.630
Dietary supplements (yes)	121 (16.7)	30 (16.9)	91 (16.7)	.961
Maintains a healthy diet (total score 0–16)	7.1±4.1	7.2±4.1	7.0±4.1	.697
Psychological distress (BSRS-5 score ≥6)	80 (11.1)	22 (12.4)	58 (10.6)	.526
Economic status (total score 0–10)	6.0±1.1	6.1±1.1	6.0±1.1	.134
Social support (total score 0–30)	24.7±4.8	25.0±4.7	24.6±4.8	.391
**Measurements**				
Systolic blood pressure (mm Hg)	131.6±21.1	131.1±18.9	131.8±21.8	.664
Diastolic blood pressure (mm Hg)	77.5±13.0	77.6±11.6	77.4±13.4	.822
Body mass index (kg/m^2^) ^a^	26.8 (24.3–29.2)	27.1 (25.0–29.5)	26.7 (24.1–29.1)	.150
Glycated hemoglobin (%) ^a^	6.2 (5.8–7.0)	6.0 (5.7–6.8)	6.3 (5.8–7.1)	.005**
Fasting sugar (mg/dL) ^a^	99.0 (90.0–117.0)	96.0 (88.0–111.0)	101.0 (91–118.8)	.066
Triglyceride (mg/dL) ^a^	126.0 (88.0–187.3)	121.0 (85.5–161.5)	128.0 (89.0–190.5)	.110
High-density lipoprotein (mg/dL)	40.3±1.0	42.2±10.6	39.4±9.6	<.001**
Low-density lipoprotein (mg/dL) ^a^	96.0 (75.0–124.0)	92.0 (74.0–116.0)	97.0 (75.0–127.3)	.022*
Total cholesterol (mg/dL) ^a^	164.5 (139.0–193.0)	162.0 (141.5–188.3)	166.0 (138.0–195.0)	.374
Hemoglobin (g/dL)	14.2±1.7	14.2±1.6	14.2±1.7	.716
eGFR (mL/min/1.73 m^2^)	85.5±23.7	89.3±20.7	81.6±24.4	<.001**
Uric acid (mg/d)	6.2±1.6	5.9±1.5	6.3±1.6	.005**
High-sensitivity CRP (mg/L) ^a^	1.3 (0.6–2.9)	1.0 (0.5-1.8)	1.4 (0.7–3.6)	.045*
**Angina characteristics**				
Severity				
Class 0 (asymptomatic)	92 (12.7)	26 (14.6)	66 (12.1)	—
Class 1	225 (31.1)	45 (25.3)	180 (33.0)	.111
Class 2	304 (42.0)	74 (41.6)	230 (42.2)	.449
Class 3	72 (10.0)	22 (12.4)	50 (9.2)	.749
Class 4	30 (4.1)	11 (6.2)	19 (3.5)	.386
Type				
Atypical	287 (39.7)	86 (48.3)	201 (36.9)	—
Typical	436 (60.3)	92 (51.7)	344 (63.1)	.007**
Frequency				
None	222 (30.7)	61 (34.3)	161 (29.5)	—
Rarely (1–3 times/month)	168 (23.2)	36 (20.2)	132 (24.2)	.172
Occasionally (1–2 times/week)	160 (22.1)	38 (21.3)	122 (22.2)	.412
Frequently (3–6 times/week)	65 (9.0)	16 (9.0)	49 (9.0)	.647
Always (≥7 times/week)	108 (14.9)	27 (15.2)	81 (14.9)	.633

^a^ Express median (interquartile range).

*p < .05. **p < .01.

**Table 2:** Summary of Participant Medical Histories

Variable	All (*n*=723)	Control (*n*=178)	Obstructive (*n*=545)	*p*
	*n* (%)/Mean±*SD*	*n* (%)/Mean±*SD*	*n* (%)/Mean±*SD*	
**Examination findings**				
Troponin I (ng/dL, *n*=238) ^a^	0 (0–2.1)	0 (0–0)	0 (0–3.7)	.044*
Creatine kinase - MB (%, *n*=231) ^a^	0 (0–3.1)	0 (0–0)	0 (0–4.0)	.193
B-type natriuretic peptide (pg/mL, *n*=107) ^a^	108.9 (41.0–394.9)	152.4 (28.2–304.4)	99.5 (42.0–423.3)	.319
Exercise electrocardiogram (positive, *n*=95)	76 (80.0)	18 (78.3)	58 (80.6)	.811
Myocardial perfusion image (positive, *n*=233)	218 (93.6)	52 (89.3)	166 (94.9)	.170
Echocardiography (abnormal, *n*=502)	192 (38.2)	28 (25.7)	164 (41.7)	.003*
Left ventricular ejection fraction (%, *n*=441)	61.6±13.3	64.6±12.5	60.3±13.4	.002*
Number of vessels with stenosis ≥50%				
0	178 (24.6)	178 (100)	—	<.001*
1	225 (31.2)	—	225 (41.3)	
2	184 (25.4)	—	184 (33.7)	
≥3	136 (18.8)	—	136 (25.0)	
Received revascularization (yes)	424 (58.6)	1 (0.6)	423 (77.6)	<.001*
**Admission diagnosis and medications (yes)**				
Diagnosis				
Angina	619 (85.6)	172 (96.6)	447 (82.0)	
Myocardial infarction	104 (14.4)	6 (3.4)	98 (18.0)	<.001*
Diuretic	81 (11.2)	17 (9.6)	64 (11.7)	.422
α-blocker	15 (2.1)	4 (2.2)	11 (2.0)	.853
β-blocker	510 (70.5)	124 (69.7)	386 (70.8)	.768
α+β-blocker	31 (4.3)	5 (2.8)	26 (4.8)	.268
Calcium channel blocker	198 (27.4)	48 (27.0)	150 (27.5)	.885
Angiotensin-converting enzyme inhibitor	114 (15.8)	11 (6.2)	103 (18.9)	<.001*
Angiotensin II receptor blocker	269 (37.2)	69 (38.8)	200 (36.7)	.620
Antiplatelet	661 (91.4)	152 (85.4)	509 (93.4)	.001*
Anticoagulation	70 (9.7)	8 (4.5)	62 (11.4)	.009*
Nitrate	217 (30.0)	35 (19.7)	182 (33.4)	.001*

^a^ Express median (interquartile range).

* *p*<.05.

### Relationship Between Angina Characteristics and CAD

Candidate risk factors for each group are shown in Table 1. Based on the results of the simple logistic regression analysis, 13 variables were identified as significantly associated with obstructive CAD. The variance inflation factor (VIF) for each variable was <2, indicating no collinearity concerns for subsequent multiple regression analysis. A Hosmer–Lemeshow test confirmed the goodness of fit of the stepwise multiple logistic regression model (*p*>.05). Multiple regression analysis revealed older age, male sex, having a history of diabetes, currently smoking, having elevated low-density lipoprotein levels, a decreased estimated glomerular filtration rate (eGFR), and having typical angina as associated with greater risk of obstructive CAD (*p*<.05). After adjusting for covariates, individuals with typical angina were found to be nearly twice as likely to have obstructive CAD than those with atypical angina (Table 3). In addition, the significant association found between typical angina and obstructive CAD persisted even after variables from cardiac examinations, for example, troponin I levels, echocardiographic parameters, and LVEF, had been incorporated into the original regression model (details not shown).

**Table 3:** Factors Contributing to Coronary Artery Disease: Stepwise Logistic Regression Analysis

Variable	*B*	*SE*	*p*	*OR*	95% CI for *OR*	
					Lower	Upper
**All (** *n* **=723)**						
Age (year)	0.04	0.02	.008^**^	1.04	1.01	1.08
Sex (male)	1.13	0.37	.002^**^	3.09	1.49	6.42
Diabetes history (yes)	0.84	0.36	.021^*^	2.32	1.14	4.72
Psychological disease history (yes)	−2.45	1.29	.058	0.09	0.01	1.09
Current smoker (yes)	1.21	0.38	.001^**^	3.37	1.60	7.09
Low-density lipoprotein (mg/dL)	0.01	0.00	.002^**^	1.01	1.01	1.02
eGFR (mL/min/1.73 m^2^)	−0.02	0.01	.009^**^	0.98	0.97	1.00
Typical angina	0.68	0.30	.025^*^	1.97	1.09	3.54
Age <65 (*n*=454)						
Age (year)	0.07	0.02	<.001^**^	1.07	1.03	1.12
Sex (male)	0.97	0.42	.021^*^	2.63	1.16	5.97
Current smoker (yes)	1.05	0.34	.002^**^	2.86	1.48	5.56
Low-density lipoprotein (mg/dL)	0.01	0.00	.025^*^	1.01	1.00	1.02
Uric acid (mg/dL)	0.20	0.10	.045^*^	1.22	1.00	1.49
High-sensitivity CRP (mg/L)	0.08	0.04	.036^*^	1.08	1.01	1.17
Typical angina	0.80	0.30	.008^**^	2.22	1.23	3.99
Age ≥65 (*n*=269)						
Diabetes history (yes)	1.95	0.59	.001^**^	7.00	2.19	22.34
Psychological disease history (yes)	−2.06	1.02	.043^*^	0.13	0.02	0.94
Angina frequency (ref=none)						
1–3 times/month	2.46	0.77	.001^**^	11.74	2.59	53.14
1–2 times/week	1.66	0.64	.010^*^	5.25	1.49	18.47
3–6 times/week	0.94	0.82	.248	2.57	0.52	12.76
≥7 times/week	1.44	1.27	.255	4.24	0.35	50.96
Females (*n* = 118)						
Diabetes history (yes)	2.27	0.63	<.001^**^	9.70	2.84	33.15
Males (*n* = 605)						
Age (year)	0.05	0.01	<.001^**^	1.05	1.02	1.08
Diabetes history (yes)	0.76	0.34	.028^*^	2.13	1.09	4.17
Psychological disease history (yes)	−1.95	0.94	.038^*^	0.14	0.02	0.89
High-density lipoprotein (mg/dL)	−0.03	0.01	.040^*^	0.97	0.94	1.00
Low-density lipoprotein (mg/dL)	0.02	0.00	<.001^**^	1.02	1.01	1.03
High-sensitivity CRP (mg/L)	0.07	0.04	.053	1.07	1.00	1.15
Typical angina	0.60	0.27	.024^*^	1.83	1.08	3.09

*Note*. The overall accuracy of the prediction model was 78.5%. In the subgroups, accuracy rates were 74.2% for age <65, 86.2% for age ≥65, 71.4% for females, and 78.9% for males. eGFR = estimated Glomerular Filtration Rate; CRP = C-reactive protein.

* *p* < .05. **p < .01.

### Subgroup Analysis by Age and Sex

As shown in Tables 4 and 5, obstructive CAD was more prevalent in participants who were older (79.2%) and male (77.5%). In this study, the participants were divided into two age groups: younger (<65 y) and older (≥65 y). The prevalence of significantly narrowed coronary arteries was >70% in each subgroup. Simple logistic regression revealed 12 variables to be significantly associated with obstructive CAD in the younger subgroup, while 9 showed significant associations in the older subgroup (details not shown). Variables showing significant correlations in the univariate analysis included angina symptom characteristics such as severity and type in the younger subgroup, and severity and frequency in the older subgroup (Table 4). The VIF values for these variables were <1.5 and <3.5 in the younger and older subgroups, respectively, indicating no collinearity issues. The Hosmer–Lemeshow test demonstrated a good fit for the multiple regression models in both subgroups. In the younger subgroup, multivariate analysis showed the risk of obstructive CAD to be 2.22 times higher in patients with typical angina than in those with atypical angina. In the older subgroup, those with an angina frequency of less than twice per week had a higher risk of obstructive CAD than those with no angina episodes during the past month (Table 3).

**Table 4:** Associations Between Angina Characteristics and Coronary Artery Disease by Age: Simple Logistic Regression Analysis

Variable	Age < 65				Age ≥ 65			
	All (*n*=454)	Control(*n*=122)	Obstructive(*n*=332)	*p*	All (*n*=605)	Control(*n*=136)	Obstructive(*n*=469)	*p*
	*n* (%)	*n* (%)	*n* (%)		*n* (%)	*n* (%)	*n* (%)	
Severity								
Class 0 (asymptomatic)	66 (14.5)	13 (10.7)	53 (16.0)	—	26 (9.7)	13 (23.2)	13 (6.1)	—
Class 1	147 (32.4)	32 (26.2)	115 (34.6)	.732	78 (29.0)	13 (23.2)	65 (30.5)	.001^*^
Class 2	193 (42.5)	55 (45.1)	138 (41.6)	.163	111 (41.3)	19 (33.9)	92 (43.2)	.001^*^
Class 3	30 (6.6)	12 (9.8)	18 (5.4)	.039^*^	42 (15.6)	10 (17.9)	32 (15.0)	.029^*^
Class 4	18 (4.0)	10 (8.2)	8 (2.4)	.004^*^	12 (4.5)	1 (1.8)	11 (5.2)	.032^*^
Type								
Atypical	185 (40.7)	61 (50.0)	124 (37.3)	—	102 (37.9)	25 (44.6)	77 (36.2)	—
Typical	269 (59.3)	61 (50.0)	208 (62.7)	.015^*^	167 (62.1)	31 (55.4)	136 (63.8)	.245
Frequency								
None	148 (32.6)	39 (32.0)	109 (32.8)	—	74 (27.5)	22 (39.3)	52 (24.4)	—
1–3 times/month	109 (24.0)	26 (21.3)	83 (25.0)	.649	59 (21.9)	10 (17.9)	49 (23.0)	.090
1–2 times/week	95 (20.9)	30 (24.6)	65 (19.6)	.378	65 (24.2)	8 (14.3)	57 (26.8)	.015^*^
3–6 times/week	38 (8.4)	10 (8.2)	28 (8.4)	.996	27 (10.0)	6 (10.7)	21 (9.9)	.457
≥7imes/week	64 (14.1)	17 (13.9)	47 (14.2)	.974	44 (16.4)	10 (17.9)	34 (16.0)	.409

* *p*<.05. ** *p <* .01.

**Table 5:** Associations Between Angina Characteristics and Coronary Artery Disease by Sex: Simple Logistic Regression Analysis

Variable	Female				Male			
	All(*n*=118)	Control(*n*=42)	Obstructive(*n*=76)	*p*	All(*n*=605)	Control(*n*=136)	Obstructive(*n*=469)	*p*
	*n* (%)	*n* (%)	*n* (%)		*n* (%)	*n* (%)	*n* (%)	
Severity								
Class 0 (asymptomatic)	11 (9.3)	9 (21.4)	2 (2.6)	—	81 (13.4)	17 (12.5)	64 (13.6)	—
Class 1	31 (26.3)	10 (23.8)	21 (27.6)	.010^*^	194 (32.1)	35 (25.7)	159 (33.9)	.570
Class 2	48 (40.7)	16 (38.1)	32 (42.1)	.009^*^	256 (42.3)	58 (42.6)	198 (42.2)	.753
Class 3	22 (18.6)	5 (11.9)	17 (22.4)	.003^*^	50 (8.3)	17 (12.5)	33 (7.0)	.101
Class 4	6 (5.1)	2 (4.8)	4 (5.3)	.060	24 (4.0)	9 (6.6)	15 (3.2)	.105
Type								
Atypical	51 (43.2)	23 (54.8)	28 (36.8)	—	236 (39.0)	63 (46.3)	173 (36.9)	—
Typical	67 (56.8)	19 (45.2)	48 (63.2)	.062	369 (61.0)	73 (53.7)	196 (63.1)	.048^*^
Frequency								
None	32 (27.1)	16 (38.1)	16 (21.1)	—	190 (31.4)	45 (33.1)	145 (30.9)	—
1–3 times/month	27 (22.9)	7 (6.7)	20 (26.3)	.063	141 (23.3)	29 (21.3)	112 (23.9)	.501
1–2 times/week	33 (28.0)	10 (23.8)	23 (30.3)	.108	127 (21.0)	28 (20.6)	99 (21.1)	.734
3–6 times/week	9 (7.6)	4 (9.5)	5 (6.6)	.769	56 (9.3)	12 (8.8)	449 (9.4)	.725
≥7 times/week	17 (14.4)	5 (11.9)	12 (15.8)	.171	91 (15.0)	22 (16.2)	69 (14.7)	.928

* *p* < .05.

Furthermore, the simple logistic regression revealed 8 and 9 variables, respectively, as significantly associated with obstructive CAD in females and males (details not shown). Variables showing significant correlations in the univariate analysis included angina symptom characteristics such as severity in females and type in males (Table 5). The VIF for these variables was <4.5 in females and <2 in males, indicating no multicollinearity issue. The Hosmer–emeshow test confirmed that the regression models were accurate (*p*>.05). In females, angina severity, type, and frequency were not significantly associated with obstructive CAD. When adjusted, males with typical angina were found to have a 1.83 times greater risk of obstructive CAD than their peers with atypical angina (Table 3).

## Discussion

To the best of the authors’ knowledge, this is the first study to concurrently characterize the type, severity, and frequency of angina symptoms associated with obstructive CAD. Moreover, this study was also the first to examine this relationship in the Taiwanese population. The findings suggest patients with typical angina symptoms face nearly twice the risk of experiencing obstructive CAD than those with atypical symptoms. However, no significant association was found between angina symptom severity and the incidence of obstructive CAD. Notably, a higher risk of obstructive CAD was observed in older participants but not younger participants who experienced infrequent or occasional angina symptoms. Therefore, the results partially support the hypothesis.

### Basic Characteristics of Participants

The ICA findings, widely accepted as the gold standard, were used in this study to define obstructive CAD (Madsen et al., 2017; Ueng et al., 2023). Previous studies using a coronary stenosis threshold of ≥50% have shown that the proportion of obstructive CAD detected by ICA ranges from 44% to 81.3% (Madsen et al., 2017; Nakas et al., 2019; Peerwani et al., 2023; Peng et al., 2021), which is within the range detected in this study (75.4%). Furthermore, the subgroup analysis in this study revealed a higher prevalence of obstructive CAD in older males, which is also consistent with previous studies (Kim et al., 2022; Peng et al., 2021; Reeh et al., 2019).

The nonanginal chest pain category was excluded in this study, and participants were categorized into two types only, as it was anticipated that reported chest pain / discomfort would be predominantly of cardiac origin. The findings showed 60.3% of participants experienced typical angina symptoms, which is similar to previous studies (Peerwani et al., 2023; Peng et al., 2021). Typically, women and older patients present with atypical angina symptoms, while specific angina symptoms are more common in patients with obstructive CAD than in those without (Peng et al., 2021). In this study, older patients (62.1%) had a slightly higher proportion of typical angina symptoms than younger patients (59.3%), which may be due to the higher prevalence of obstructive CAD in older patients (Peng et al., 2021). Reeh et al., 2019 reported that, compared with women, men had a significantly higher proportion of typical angina symptoms, which was similar to this study (61% in men and 56.8% in females).

### Relationship Between Angina Characteristics and CAD

Prior research indicating that individuals who experience typical angina symptoms face a 1.5–3.5 times higher risk of obstructive CAD (Chinnaiyan et al., 2012; Madsen et al., 2017; Nakas et al., 2019; Peng et al., 2021) was confirmed in this study (*OR*=1.97). Almost half of the participants in this study with nonobstructive CAD showed typical angina symptoms, likely due to coronary microcirculatory dysfunction unrelated to significant stenosis in their epicardial arteries (Nakas et al., 2019). Moreover, one-third of the participants with obstructive CAD did not have typical angina symptoms. These cases may include atypical angina, nonanginal chest pain, or even asymptomatic individuals. The findings confirm that specific angina symptoms are significantly associated with an increased risk of obstructive CAD, even after taking various risk factors and a history of psychological disease into account. It is important to note that the probability of typical angina symptoms predicting obstructive CAD is influenced by age and sex, as indicated by a previous pooled analysis (Knuuti et al., 2020). In this study, a significant relationship was identified between typical angina symptoms and obstructive CAD in the younger age and male subgroups only. This may be because older individuals and females are more likely to have atypical symptoms such as microvascular dysfunction and epicardial coronary artery spasm, which often occur in nonobstructive CAD (Peng et al., 2021; Ueng et al., 2023). However, it is essential to note that the subgroup analysis in this study was conducted using small sample sizes, rendering definitive conclusions impossible. Future studies using larger cohorts will be necessary to validate these findings.

In the CONSERVE study, which utilized machine learning models to explore factors associated with obstructive CAD, angina severity was found to be among the top three predictive variables (Baskaran et al., 2020). Another study identified worse CCSC categories as associated with higher atherosclerotic burden in terms of angiographic score (Guimaraes et al., 2021). However, the results of this study did not show a significant correlation between the angina symptom severity and obstructive CAD, potentially because angina is subjective, with each person having a different threshold for experiencing chest pain/discomfort. This threshold is influenced by a complicated interplay of physical, psychological, and social factors (Zimmerman et al., 2016). Paradoxically, angina may present with symptoms elicited by strenuous exercise or emotional stress. However, angina at rest or with minimal activity often indicates an acute coronary syndrome. Those with obstructive CAD may also exhibit atypical symptoms such as dyspnea or fatigue (Gulati et al., 2021), and the heart’s various protective mechanisms allow angina symptoms to diminish with further (walk-through angina) or subsequent exercise (warm-up angina; Knuuti et al., 2020). Lastly, the relationship between angina and obstructive CAD may be influenced when CAD coexists with conditions that may produce angina-like symptoms (e.g., heart failure; Knuuti et al., 2020).

Angina symptom threshold can vary significantly from day to day and even within a single day (Knuuti et al., 2020). Guimaraes et al. (2021) found angina stability and frequency, as measured by the SAQ, to be unrelated to atherosclerotic burden scores. In this research, the frequency of angina symptoms was found to be significantly linked only to obstructive CAD in the older age group. Although the wide confidence interval in this study potentially affects the precision of the results, health care providers should remain vigilant for rare or occasional angina symptoms in older patients.

### Implications for Research and Practice

The aim of this research was to expand upon various traditional risk factors, focusing on psychosocial factors that independently predict angina symptoms (Schef et al., 2023; C. C. Tsai, Chuang, et al., 2019). These factors, entered into the Diamond and Forrester models, were used to form a predictive model for angina symptoms and obstructive CAD. The findings indicate that the type of angina symptom is more important than either symptom severity or frequency. Health care professionals should monitor angina symptoms closely in patients with suspected or known CAD. Moreover, the results of this study demonstrate that the predictive value of typical angina symptoms varies based on age and sex, underscoring the need for further research. Symptoms may display distinct pretest probabilities for disease across low-prevalence settings (e.g., general practice), medium-prevalence settings (e.g., the emergency department), and high-prevalence settings (e.g., the coronary care unit; Bruyninckx et al., 2008). In future studies, the estimated pretest probability of angina symptoms concerning obstructive CAD should be established and compared across different prevalence settings.

Finally, obtaining a patient’s symptom history can assist health care professionals in identifying potential issues and establishing a baseline for evaluating the effectiveness of treatment in the future (Alkhaqani, 2023). Assessments represent an autonomous responsibility of nurses, and evaluating angina symptoms is a fundamental competence of cardiac nurses (Bagnasco et al., 2021). The availability and use of concise tools can help integrate patient-reported outcomes into the clinical workflow. A structured AEF can standardize the collection process and enhance the accuracy of angina symptom data, which may then be integrated into electronic medical records or linked to administrative data sources for future long-term tracking and comparison.

### Strengths and Limitations

The strengths of this study include the development of the AEF questionnaire, which may be used in both future research and clinical practice to enhance medical staff recognition of related symptoms (Kemp et al., 2019; Shafiq et al., 2016). However, several limitations of this study must be noted. The cross-sectional design limited the establishment of causal relationships between variables, and the purposive sampling approach may have led to sampling bias. Using questionnaires for self-reported symptoms increases the risk of recall and reporting biases. The classification and prevalence of angina symptoms and obstructive CAD may be influenced by variations in examination methods or cutoff points, potentially influencing the strength of the observed relationships. Although we attempted to adjust for confounding variables, factors such as medication usage for symptom relief were not considered. Furthermore, this study was conducted at a single center in northern Taiwan, a region with a high prevalence of CAD, limiting the generalizability of the findings to other areas or populations.

### Conclusions

In this study, the nature of typical angina symptoms, rather than their severity or frequency, was shown to be significantly associated with a higher risk of obstructive CAD in patients who undergo coronary angiography. Evaluating these angina symptoms may enhance risk assessment as part of a comprehensive management strategy for patients with known or suspected CAD. However, it is essential to note that these results require further validation using larger-scale prospective studies. Furthermore, developing an accurate predictive model for diagnosing obstructive CAD will be crucial to advancing future research in this field.

## Supplementary Material
