# Supplementary material for: Association Between Angina Symptom Characteristics and Obstructive Coronary Artery Disease: A Comparative Cross-Sectional Study
Source: J Nurs Res. 2025 Aug 4;33(5):e410. doi: 10.1097/jnr.0000000000000693 (PMC12466170; doi:10.1097/jnr.0000000000000693)
Supplement: Supplementary file 1 [file jnr-33-e410-s001.docx]

**Appendix**

Angina Evaluation Form

1. Have you ever experienced chest pain or discomfort?

□ No

□ Yes (Please continue to answer the following questions)

1. In which of the following situations do you usually feel chest pain or discomfort?

□ With effort exertion (e.g., moving heavy things, lifting, or rapid/prolonged strenuous activity)

□ With moderate exertion or emotional stress (e.g., agitation, after heavy meals, cold weather, fast walking on flat ground, climbing hills, or climbing two or more floors)

□ With mild exertion (e.g., walking 100–200 meters at a normal pace or climbing one flight of stairs)

□ At rest

1. What do you usually do when you feel chest pain or discomfort while walking?

- Stop walking □ Walk slower □ Keep walking at the same speed

1. When you feel chest pain or discomfort while walking, if you stop moving, does the pain or discomfort usually disappear?

□ Yes □No

1. How long does it usually take for the pain or discomfort to disappear when you feel it?

□ Less than 1 minute □ 1–10 minutes □ 11–20 minutes

□ 21–30 minutes □ More than 30 minutes

1. Where does your chest pain or discomfort usually occur?

□ Right upper chest □ Middle-upper chest □ Left upper chest

□ Right lower chest □ Middle-lower chest □ Left lower chest

1. How often did you feel chest pain or discomfort in the past month?

□ None □ Rarely (1–3 times/month)

□ Occasionally (1–2 times/week) □ Frequently (3–6 times/week)

□ Always (≥ 7 times/week)
